# Antiphospholipid Antibodies Bind ATP: A putative Mechanism for the Pathogenesis of Neuronal Dysfunction

**DOI:** 10.1080/17402520500217844

**Published:** 2005-09

**Authors:** J. Chapman, L. Soloveichick, S. Shavit, Y. Shoenfeld, A. D. Korczyn

**Affiliations:** 1 Department of Physiology and Pharmacology Sackler Faculty of Medicine Tel Aviv University Ramat Aviv 69978 Israel; 2 Department of Neurology Sackler Faculty of Medicine Tel Aviv University Ramat Aviv 69978 Israel; 3 Department of Biochemistry Sackler Faculty of Medicine Tel Aviv University Ramat Aviv 69978 Israel; 4 Department of Medicine B and Research Unit of Autoimmune Diseases Sheba Medical Center Tel Hashomer Israel

## Abstract

Antiphospholipid antibodies (aPL) generated in experimental animals cross-react with ATP. We therefore examined the possibility that aPL IgG from human subjects bind to ATP by affinity column and an enzyme linked immunosorbent assay (ELISA). Sera with high levels of aPL IgG were collected from 12 patients with the antiphospholipid syndrome (APS). IgG fractions from 10 of 12 APS patients contained aPL that could be affinity-bound to an ATP column and completely eluted with NaCl 0.5 M. A significant (>50%) inhibition of aPL IgG binding by ATP 5 mM was found in the majority. Similar inhibition was obtained with ADP but not with AMP or cAMP. All the affinity purified anti-ATP antibodies also bound β_2_-glycoprotein-I (β_2_-GPI, also known as apolipoprotein H) suggesting that, similar to most pathogenic aPL, their binding depends on this serum cofactor. We further investigated this possibility and found that the binding of β_2_-GPI to the ATP column was similar to that of aPL IgG in that most was reversed by NaCl 0.5 M. Furthermore, addition of β_2_-GPI to aPL IgG significantly increased the amount of aPL binding to an ATP column. We conclude that aPL IgG bind ATP, probably through β_2_-GPI. This binding could interfere with the normal extracellular function of ATP and similar neurotransmitters.


*Clinical & Developmental Immunology*, September 2005; 12(3): 175–180

Taylor & Francis

Taylor & Francis Group

### Antiphospholipid antibodies bind ATP: A putative mechanism for the pathogenesis of neuronal dysfunction

J. CHAPMAN[1,2,3], L. SOLOVEICHICK[1], S. SHAVIT[1], Y. SHOENFELD[4], & A.D. KORCZYN[1,2]

[1]*Department of Physiology and Pharmacology, Sackler Faculty of Medicine, Tel Aviv University, Ramat Aviv 69978, Israel,* [2]*Department of Neurology, Sackler Faculty of Medicine, Tel Aviv University, Ramat Aviv 69978, Israel,* [3]*Department of Biochemistry, Sackler Faculty of Medicine, Tel Aviv University, Ramat Aviv 69978, Israel, and* [4]*Department of Medicine B and Research Unit of Autoimmune Diseases, Sheba Medical Center, Tel Hashomer, Israel*

**Abstract**
Antiphospholipid antibodies (aPL) generated in experimental animals cross-react with ATP. We therefore examined the possibility that aPL IgG from human subjects bind to ATP by affinity column and an enzyme linked immunosorbent assay (ELISA). Sera with high levels of aPL IgG were collected from 12 patients with the antiphospholipid syndrome (APS). IgG fractions from 10 of 12 APS patients contained aPL that could be affinity-bound to an ATP column and completely eluted with NaCl 0.5 M. A significant (>50%) inhibition of aPL IgG binding by ATP 5 mM was found in the majority. Similar inhibition was obtained with ADP but not with AMP or cAMP. All the affinity purified anti-ATP antibodies also bound $\beta_2$-glycoprotein-I ($\beta_2$-GPI, also known as apolipoprotein H) suggesting that, similar to most pathogenic aPL, their binding depends on this serum cofactor. We further investigated this possibility and found that the binding of $\beta_2$-GPI to the ATP column was similar to that of aPL IgG in that most was reversed by NaCl 0.5 M. Furthermore, addition of $\beta_2$-GPI to aPL IgG significantly increased the amount of aPL binding to an ATP column. We conclude that aPL IgG bind ATP, probably through $\beta_2$-GPI. This binding could interfere with the normal extracellular function of ATP and similar neurotransmitters.

**Keywords:** *Antiphospholipid antibodies, adenosine triphosphate, affinity columns, beta2-glycoprotein-I*

### Introduction

Antiphospholipid antibodies (aPL) are defined as immunoglobulins which bind anionic phospholipids. The occurrence of such antibodies, especially IgG aPL, in patients with clinical manifestations such as thromboembolic disease and recurrent abortions, defines the antiphospholipid syndrome (APS, or Hughes' syndrome) (Hughes 1985, Asherson et al. 1996). A central issue in the study of these antibodies has been their antigenic specificity. The antibodies by which this syndrome was named were initially measured by binding to cardiolipin (Harris et al. 1983). Subsequently, it was found that they cross-react with other phospholipids such as phosphatidylserine (Gilburd et al. 1993) as well as with DNA (Asherson and Shoenfeld 1994) which is rich in phosphate groups. Some aPL bind endothelial cells (Del Papa et al. 1995, Dueymes et al. 1996, Bordron et al. 1998). A crucial observation has been that aPL, including monoclonal antibodies, cross-react with other relevant protein antigens such as $\beta_2$-glycoprotein-I ($\beta_2$GPI, also known as apolipoprotein H) (Galli et al. 1990, Shoenfeld and Meroni 1992). This protein may serve as a cofactor necessary for the binding of aPL to phospholipids and related antigens such as endothelial cells.

In addition to phospholipids, aPL bind other antigens such as adenosine triphosphate (ATP). This phenomenon has been described with monoclonal and polyclonal aPL induced in experimental animals which have been shown to bind to ATP utilizing a lyposome based assay (Wassef et al. 1984, Alving et al. 1987, Wassef et al. 1993). These reports are of special

Correspondence: J. Chapman, Department of Physiology and Pharmacology, Sackler Faculty of Medicine, Tel Aviv University, Ramat Aviv 69978, Israel. Tel: 972 3 640 8734. Fax: 972 3 640 9113. E-mail: jchapman@post.tau.ac.il

ISSN 1740-2522 print/ISSN 1740-2530 online © 2005 Taylor & Francis
DOI: 10.1080/17402520500217844

interest in view of the central role of ATP in many biochemical reactions as well as its well-established role as a neurotransmitter (Vizi et al. 1997). However, since the aPL reacting with ATP were derived experimentally in animals their relevance to human disease is unknown. We now present evidence that aPL IgG from human APS patients also bind ATP and that such binding may involve the serum cofactor $\beta_2$GPI.

### Methods

All subjects had high serum levels of aPL IgG (>20 GPLU) by a standard assay based on the method of Harris (Harris 1990) and had at least thrombosis or recurrent abortions as well as neurological manifestations. Subjects included 12 patients with APS aged 32–72 years who did not fulfill the American Rheumatism Association criteria for systemic lupus erythematosus (SLE). All patients were treated with aspirin, 6 were treated with oral anticoagulants and none received immunosupressive drugs. In view of the potential relevance of ATP binding antibodies to the brain we included patients with involvement of the central nervous system such as dementia or stroke. Dementia was diagnosed by DSM-IV (Association AP 1994) criteria and supported by cognitive examinations in seven of the APS patients. The remaining subjects without dementia had strokes. Controls were age matched without immunological or neurological disease ($n = 10$). All samples were obtained by informed consent as approved by the Tel Aviv University ethics committee.

The ELISA method used for the measurement of aPL was similar to that previously described (Harris 1990) except for some modifications: Phospholipids were obtained from Sigma (St Louis, MO). Coating of antigen (100 µg/ml) in ethanol (for cardiolipin (CL)) or chloroform/methanol 1:5 (v:v, for phosphatidic acid (PA), phosphatidylinositol (PI), phosphatidylserine (PS), phosphatidylcholine (PC) and phosphatidylethanolamine (PE)) was performed by allowing samples to evaporate overnight at 4°C in Linbro-Flow (ICN Biomedicals, CA) polystyrene ELISA plates. In the ELISA for anti-$\beta_2$GPI antibodies, the plates were coated with $\beta_2$GPI (prepared by the method of Polz et al. (1980), 1 µg/well) diluted in phosphate buffered saline (PBS) overnight at 4°C. Non-specific binding to the plates was blocked with 1% bovine serum albumin (BSA) in PBS for 2 h. The plates were then washed (×10) with water, and patients sera were incubated overnight in PBS with 5% fetal calf serum (FCS, as a source of $\beta_2$GPI). In some tests, FCS was omitted in order to test the serum dependence of the antibody binding. In competition assays, the competing substance was added to the diluted serum sample 10 min prior to incubation in the plate. After washing the plates, bound human IgG was measured by means of an alkaline phosphatase conjugated second antibody (Sigma, St Louis, MO) using p-nitrophenyl phosphate as a substrate, and absorbance was read at 405 nm in each well after 30 min. All samples were tested in duplicate or triplicate on antibody coated wells and on wells coated with ethanol or chloroform/methanol or PBS. For each sample, specific binding was calculated by subtracting the values obtained in the wells without antigen from those obtained in antigen-coated wells.

Purification of IgG was essential in order to ensure that serum-derived ATPase activity did not destroy the ligand in the ATP affinity column experiments. The purification was performed by standard procedures on Protein-G columns (Zymed). In brief, filtered serum was applied to the column diluted 1:3 in $Na_2HPO_4$ 0.02M, NaCl 0.15M, pH 7.0 (sodium phosphate buffer). The column was then washed (10 volumes) with sodium phosphate buffer. IgG, bound to the column, was eluted with 0.1 M Acetic acid, pH 2.8, and the pH of collected fractions was immediately neutralized with Tris HCl 1 M, pH 8.0. Purified IgG was quantified by measuring absorbance at 280 nm and its aPL content was assayed by ELISA as described above.

Affinity purification of ATP-binding IgG was performed on a 1 ml ATP-agarose column (activation by cyanogen bromide, attachment of 2–10 µmoles ATP at C-8, Sigma A2767). Purified IgG fractions were passed three times through the column (10 mM Tris, pH = 8.0). Elution was performed with 10 ml each of 100, 200, 300, 400 and 500 mM NaCl. In some samples, further elution was attempted by 10 ml each of 100 mM acetate pH = 2.8 and 100 mM triethylamine, pH = 11.5. The pH of the fractions was immediately neutralized with 1M Tris pH = 8.0 and the absorbance at 280 nm was determined. Relevant fractions were then assayed by ELISA for aPL IgG. When not in use the columns were stored in PBS with 0.1% sodium azide.

### Results

Binding of antibodies to ATP was demonstrated by affinity purification on ATP columns. Purification of anti-ATP antibodies from the sera of two APS patients (aged 68 and 72) with dementia are presented in Figures 1 and 2. In each patient, fractions collected from the column were assayed for IgG content by spectroscopy (280 nm) and by anti-cardiolipin (aCL) ELISA as a measure of aPL IgG content. As can be seen, in the two patients (Figures 1 and 2) a significant amount of IgG bound to the column and was eluted with 500 mM NaCl. No additional IgG was eluted at pH 2.8 or 11.5 (Figure 2). In these two patients, all aPL IgG were contained in the IgG eluted from the column while the IgG which was not bound by the column did not contain aPL IgG. In 10 of the 12

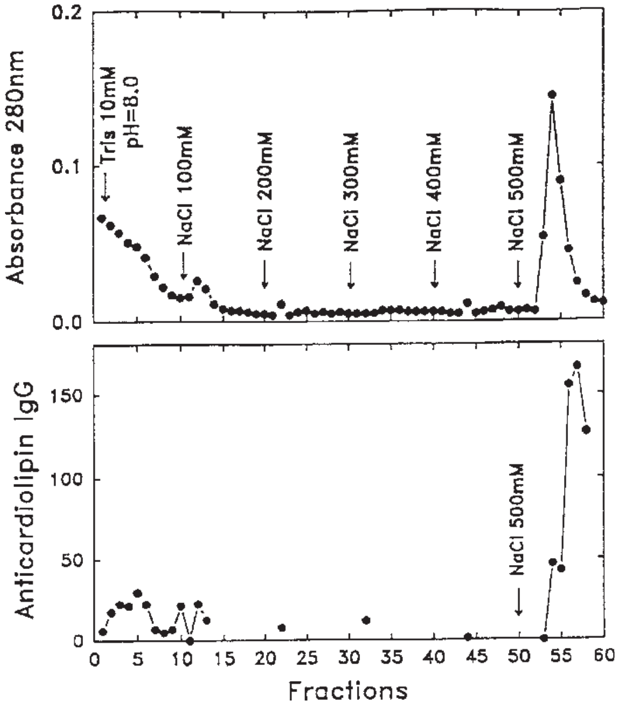

Figure 1. Results of affinity purification of anti-ATP IgG from a demented APS patient performed as described in the text. Elution buffers are indicated. Fractions collected from the column were assayed for total IgG content by spectroscopy (280 nm, upper panel) and for anticardiolipin antibodies (aCL) by ELISA (lower panel). The results indicate that most aCL could be eluted by high salt (500 mM NaCl).

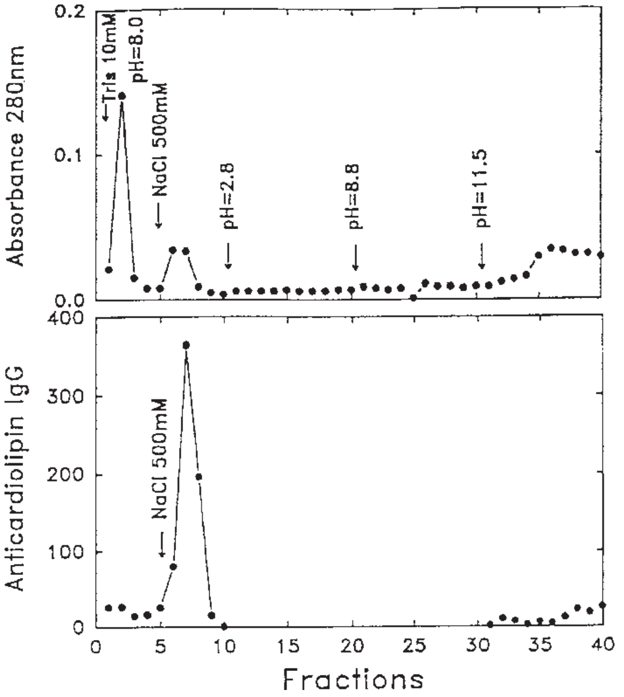

Figure 2. Results of affinity purification of anti-ATP IgG from a demented APS patient performed as described in the text. Elution buffers are indicated. Fractions collected from the column were assayed for total IgG content by spectroscopy (280 nm, upper panel) and for anticardiolipin IgG (aCL) by ELISA (lower panel). The results indicate that most aCL could be eluted by the high salt (500 mM NaCl) concentration and that no additional antibody was eluted by pH changes.

patients examined over 60% of the aPL could be affinity bound by the ATP column. In the two remaining APS patients, one with dementia and one with stroke, less than 40% of aPL bound to ATP. There were no significant differences between the demented and non-demented patients. Control IgG with no aPL did not bind to ATP to any measurable degree.

All competition assays were performed with either PA or CL as antigens and since similar results were obtained with both these antigens, those with PA are presented in Figures 3 and 4. Figure 3 presents the binding to PA of antibodies from the serum (dilutions 1:400 and 1:800) of a 60-year-old APS patient (with a history of recurrent abortions and dementia) in the presence of 0–10 mM of ATP. As can be seen, at 4–6 mM ATP a significant inhibition of binding to PA was observed at both serum dilutions. In order to examine whether other phosphorylated purines may also inhibit binding of this serum (dilution 1:800) we utilized 5 mM concentrations of ATP, adenosine diphosphate (ADP), adenosine monophosphate (AMP) and cyclic-AMP (cAMP). Results from such an inhibition ELISA are presented in Figure 4 which demonstrates the greatest inhibition with ATP and ADP, less with AMP and none with cAMP. Similar results were obtained with IgG from 4 additional APS

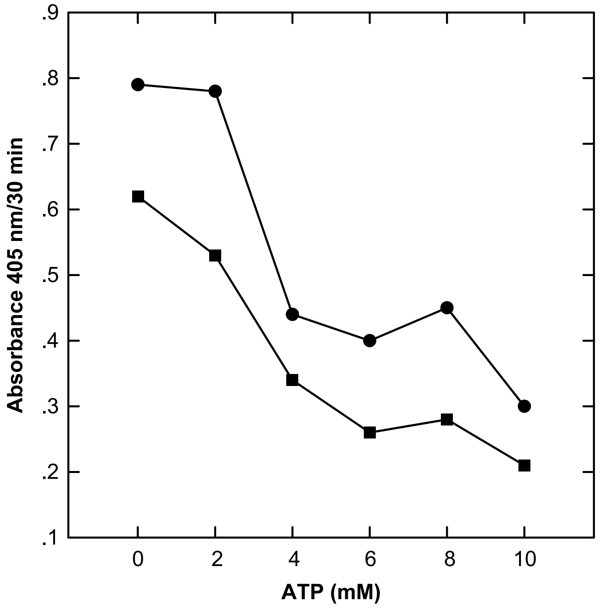

Figure 3. The binding to PA of antibodies from the serum (dilutions 1:400 and 1:800) of an APS patient in the presence of 0–10 mM of ATP. The results indicate that significant inhibition of antibody binding was achieved at concentrations exceeding 4 mM ATP.

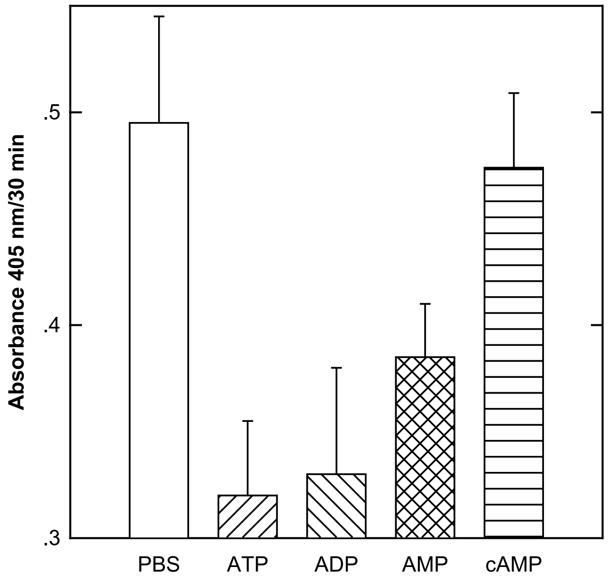

Figure 4. The binding of IgG to PA, measured in the serum of an APS patient (diluted 1:400 in phosphate buffered saline, PBS) and in the presence of 5 mM concentrations of ATP, ADP, AMP and cAMP. The results are the means ± SE of experiments performed in triplicate with one representative serum (of 5 tested).

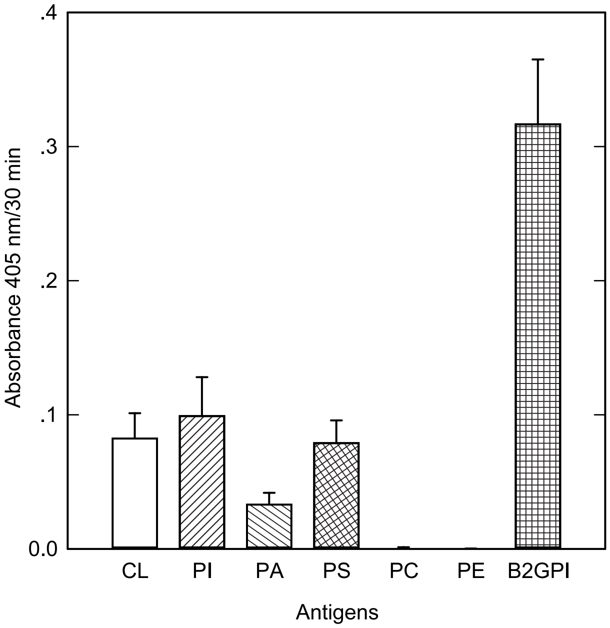

Figure 5. The antigenic specificity of IgG that was bound by an ATP column. Binding of IgG to CL, PI, PA, PS, PC, PE and $\beta_2$-Glycoprotein I ($\beta_2$GPI) were measured in samples obtained from 8 patients as described in the methods. Results are presented as mean ± SE.

patients (not shown). In IgG that bound the ATP column, aPL binding (measured to CL) could also be inhibited significantly by 5 mM ATP in IgG (61 ± 4% inhibition, mean ± SE). In contrast, aPL IgG that was not bound by the ATP column was significantly less inhibited by 5 mM ATP (25 ± 12% inhibition, $p < 0.01$, paired *t*-test).

Since binding of aPL generally depends on the presence of the serum protein $\beta_2$GPI, the serum dependency of the ATP-affinity purified aPL was examined in samples from 8 patients. All aPL, either bound or not bound by the ATP column, were completely dependent on the presence of serum for binding to cardiolipin (less than 2% of the signal in the absence of FCS). Further examination of IgG from the same 8 patient samples eluted from the ATP column revealed that they bound similarly to negatively charged phospholipids such as CL, PS, PI and PA but not to the neutral phospholipids PC and PE (Figure 5). High levels of binding to the protein cofactor $\beta_2$GPI were measured in all the samples (Figure 5) providing direct evidence for the interaction of ATP-binding aPL and this protein.

The serum dependence and β2GPI binding properties of the ATP binding IgG raised the possibility that IgG binding to the ATP column may involve complexes of IgG with $\beta_2$GPI. This possibility was examined by a series of experiments in which the binding of $\beta_2$GPI to ATP was measured. As can be seen in a representative experiment (Figure 6), about half of the $\beta_2$GPI applied bound to the ATP column and could be eluted by 500 mM NaCl. In additional experiments we found that 300 mM NaCl was sufficient to elute all $\beta_2$GPI from the ATP column (not shown). Figure 6 also presents the amount of IgG from one patient bound by the ATP column. As can bee seen, there was a 4 fold increase in the amount of

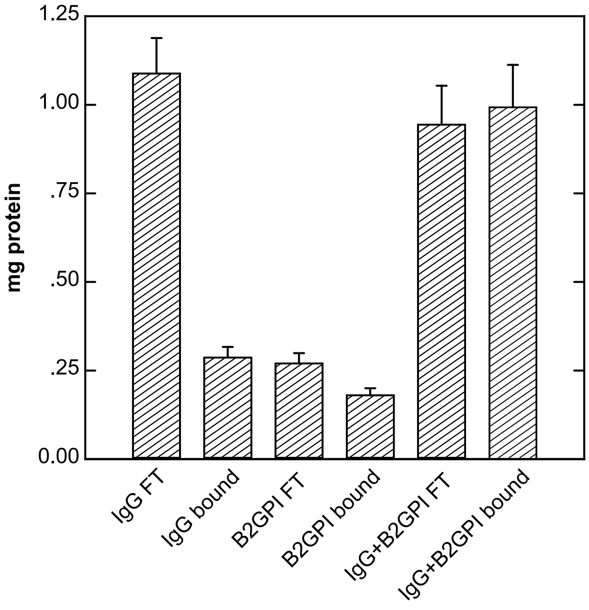

Figure 6. The influence of $\beta_2$GPI on the binding of IgG to an ATP column. IgG (1.5 mg) from a demented APS patient with high levels of aCL and $\beta_2$GPI (0.5 mg) were applied separately or in combination to an ATP column. Fractions that flowed through the column (FT) or were bound by the column (bound) were collected and assayed for protein as described in the Methods. All protein could be eluted from the column with 500 mM NaCl.

protein bound by the ATP column when the IgG was applied to the ATP column together with $\beta_2$GPI. The increased amount of protein bound to the column could only partially be accounted for by $\beta_2$GPI and therefore represents a 2–3 fold increase in the amount of IgG bound to the column.

### Discussion

The main finding in the present study was that aPL IgG from many APS patients also bind ATP. This was established by both direct binding and competition assays. Though the competition assay results may indicate specific cross-reactivity with ATP, they may also be non-specific. The co-purification of anti-ATP and aPL IgG by affinity chromatography in most patients indicates a specific IgG-ATP interaction and this interpretation is strengthened by the identification in several patients of aPL–IgG which do not bind ATP. The anti-ATP IgG were very common in the group of APS patients examined and there was no indication that they were specifically associated with dementia or with advanced age. A more detailed examination of the clinical correlates of these antibodies will necessitate significantly larger groups of patients with different manifestations of APS as well as relevant controls.

In this study, as well as in previous studies in rats (Wassef et al. 1984, Wassef et al. 1993), aPL IgG binding could be inhibited by a range of phosphorylated compounds. Other workers have shown cross-reactivity of aPL antibodies with DNA (Eilat et al. 1986, Smeenk et al. 1987). Furthermore, the "true" antigen bound by aPL–IgG is now under intense debate (Galli 1996, Roubey 1996), especially regarding the role of $\beta_2$GPI which is essential for the binding of aPL–IgG in the standard ELISA assay (Galli et al. 1990). In our experiments, none of the affinity purified anti-ATP IgG bound zwitterionic phospholipids such as PC or PE and their binding to CL was totally dependent on the presence of serum. These findings raised the possibility that aPL were affinity bound to ATP through a protein cofactor such as $\beta_2$GPI, and this hypothesis is supported by the significant binding of $\beta_2$GPI to the ATP column which could be completely reversed by the same concentrations of NaCl used to elute anti-ATP antibodies. Adding $\beta_2$GPI to an IgG sample increased the amount of IgG bound to the ATP column (Figure 6), although it is not clear whether this is the results of enhanced binding of IgG, $\beta_2$GPI, or both, to the column. A plausible explanation for the binding of aPL to the ATP column is through the presence of $\beta_2$GPI or other proteins in the purified IgG preparations. Though these preparations are highly enriched in IgG, any proteins complexed with these antibodies are co-purified in the procedure. The presence of $\beta_2$GPI has been recorded in immune complexes from patients with SLE (George et al. 1999) and in preliminary studies we have found a band corresponding to $\beta_2$GPI in protein G purified IgG from APS patients. Further studies are needed to substantiate this hypothesis.

aPL IgG cross-react with many antigens, but their pathogenic potential depends on several factors including their concentration and the availability of relevant antigens. Such considerations may pertain to the potential functional significance of the present results: At the respective levels of intracellular ATP (5 mM) and IgG (less than 1/100 serum concentration) ATP may inhibit aPL IgG binding as found *in vitro* in the present study. It is tempting to suggest that the intracellular concentration of ATP in lymphocytes enables them to secrete aPL IgG in spite of the ability of these antibodies to bind cell phospholipids. In contrast, in the extracellular space the concentration of ATP is in the μM range and the higher concentrations of ATP-binding IgG in some patients may limit ATP from functioning as a neurotransmitter binding to purinergic receptors (Vizi et al. 1997). Thus these antibodies, by binding to ATP or other purinergic transmitters such as ADP, may modulate their effect.

High levels of circulating aPL IgG have been implicated in neurological diseases such as focal cerebral ischemia, myelopathy, Guillain–Barre syndrome, migraine, chorea, epilepsy and dementia (Lubbe and Walker 1983, Coull et al. 1987, Levine et al. 1987, Inzelberg and Korczyn 1989, Inzelberg et al. 1992, Herranz et al. 1994, Levine et al. 1995, Cervera et al. 1997). The pathogenesis of nervous system disease associated with aPL IgG is still under investigation. Much of the effort has been aimed at evaluating these antibodies as a risk factor for stroke (Coull et al. 1987, Levine et al. 1995). However, the ubiquitous distribution of phospholipids provides a theoretical basis both for vascular lesions and for direct damage to the brain. aPL IgG have been shown to bind to brain vascular endothelium, platelet membranes, neuronal membranes and myelin sheaths, and may consequently alter neural structure and function directly or indirectly (Del Papa et al. 1995, Fanelli et al. 1997). Permeabilization of synaptic structures by direct binding of aPL has recently been proposed as a pathogenetic mechanism in APS (Chapman et al. 1999). This may be relevant since several neurological manifestations cannot be explained as consequences of vascular damage. A further possibility worth consideration is that aPL antibodies bind neurotransmitters. The results of the present study may provide the basis for establishing such pathogenetic mechanisms in APS involving the nervous system.

### Acknowledgements

This study was supported by a Fellowship to JC from the National Institute for Psychobiology in Israel, The Kass foundation of the American Physicians for Israel, the Schreiber foundation, the Sieratzki Chair of Neurology, Tel Aviv University, the Miriam Turjanski de Gold and Dr. Roberto Gold Fund for Neurological Research, and the Streifler Foundation for Neurological Research.

### References

Alving BM, Banerji B, Fogler WE, Alving CR. 1987. Lupus anticoagulant activities of murine monoclonal antibodies to liposomal phosphatidylinositol phosphate. Clin Exp Immunol 69:403–408.

Asherson RA, Shoenfeld Y. 1994. The significance of antibodies to DNA in the primary antiphospholipid syndrome [editorial]. Clin Exp Rheumatol 12:1–3.

Asherson RA, Cervera R, Piette J-C, Shoenfeld Y. 1996. The antiphospholipid syndrome. Boca Raton, FL: CRC Press.

Association AP. 1994. Diagnostic and statistical manual of mental disorders: DSM-IV. 4th edn. Washington, DC: American Psychiatric Association.

Bordron A, Dueymes M, Levy Y, Jamin C, Ziporen L, Piette JC, Shoenfeld Y, Youinou P. 1998. Anti-endothelial cell antibody binding makes negatively charged phospholipids accessible to antiphospholipid antibodies. Arthritis Rheum 41:1738–1747.

Cervera R, Asherson RA, Font J, Tikly M, Pallares L, Chamorro A, Ingelmo M. 1997. Chorea in the antiphospholipid syndrome. Clinical, radiologic, and immunologic characteristics of 50 patients from our clinics and the recent literature. Medicine (Baltimore) 76:203–212.

Chapman J, Cohen-Armon M, Shoenfeld Y, Korczyn AD. 1999. Antiphospholipid antibodies permeabilize and depolarize brain synaptoneurosomes. Lupus 8:127–133.

Coull BM, Bourdette DN, Goodnight Jr, SH, Briley DP, Hart R. 1987. Multiple cerebral infarctions and dementia associated with anticardiolipin antibodies. Stroke 18:1107–1112.

Del Papa N, Guidali L, Spatola L, Bonara P, Borghi MO, Tincani A, Balestrieri G, Meroni PL. 1995. Relationship between anti-phospholipid and anti-endothelial cell antibodies III: Beta 2 glycoprotein I mediates the antibody binding to endothelial membranes and induces the expression of adhesion molecules. Clin Exp Rheumatol 13:179–185.

Dueymes M, Levy Y, Ziporen L, Jamin C, Piette JC, Shoenfeld Y, Youinou P. 1996. Do some antiphospholipid antibodies target endothelial cells? Ann Med Interne (Paris) 147 Suppl. 1:22–23.

Eilat D, Zlotnick AY, Fischel R. 1986. Evaluation of the cross-reaction between anti-DNA and anti-cardiolipin antibodies in SLE and experimental animals. Clin Exp Immunol 65:269–278.

Fanelli A, Bergamini C, Rapi S, Caldini A, Spinelli A, Buggiani A, Emmi L. 1997. Flow cytometric detection of circulating activated platelets in primary antiphospholipid syndrome. Correlation with thrombocytopenia and anticardiolipin antibodies. Lupus 6:261–267.

Galli M, Comfurius P, Maassen C, Hemker HC, de Baets MH, van Breda-Vriesman PJ, Barbui T, Zwaal RF, Bevers EM. 1990. Anticardiolipin antibodies (ACA) directed not to cardiolipin but to a plasma protein cofactor. Lancet 335:1544–1547.

Galli M. 1996. Non beta 2-glycoprotein I cofactors for antiphospholipid antibodies. Lupus 5:388–392.

George J, Gilburd B, Langevitz P, Levy Y, Nezlin R, Harats D, Shoenfeld Y. 1999. Beta2 glycoprotein I containing immune-complexes in lupus patients: Association with thrombocytopenia and lipoprotein (a) levels. Lupus 8:116–120.

Gilburd B, Stein M, Tomer Y, Tanne D, Abramski O, Chapman Y, Ahiron A, Blank M, Shoenfeld Y. 1993. Autoantibodies to phospholipids and brain extract in patients with the Guillain–Barre syndrome: Cross-reactive or pathogenic? Autoimmunity 16:23–27.

Harris EN, Gharavi AE, Boey ML, Patel BM, Mackworth-Young CG, Loizou S, Hughes GR. 1983. Anticardiolipin antibodies: Detection by radioimmunoassay and association with thrombosis in systemic lupus erythematosus. Lancet 2:1211–1214.

Harris EN. 1990. Special report. The second international anti-cardiolipin standardization workshop/the kingston anti-phospholipid antibody study (KAPS) group. Am J Clin Pathol 94:476–484.

Herranz MT, Rivier G, Khamashta MA, Blaser KU, Hughes GR. 1994. Association between antiphospholipid antibodies and epilepsy in patients with systemic lupus erythematosus. Arthritis Rheum 37:568–571.

Hughes GR. 1985. The anticardiolipin syndrome. Clin Exp Rheumatol 3:285–286.

Inzelberg R, Korczyn AD. 1989. Lupus anticoagulant and late onset seizures [see comments]. Acta Neurol Scand 79:114–118.

Inzelberg R, Bornstein NM, Reider I, Korczyn AD. 1992. The lupus anticoagulant and dementia in non-SLE patients. Dementia 3:140–145.

Levine SR, Joseph R, G DA, Welch KM. 1987. Migraine and the lupus anticoagulant. Case reports and review of the literature. Cephalalgia 7:93–99.

Levine SR, Brey RL, Sawaya KL, Salowich-Palm L, Kokkinos J, Kostrzema B, Perry M, Havstad S, Carey J. 1995. Recurrent stroke and thrombo-occlusive events in the antiphospholipid syndrome. Ann Neurol 38:119–124.

Lubbe WF, Walker EB. 1983. Chorea gravidarum associated with circulating lupus anticoagulant: Successful outcome of pregnancy with prednisone and aspirin therapy. Case report. Br J Obstet Gynaecol 90:487–490.

Polz E, Wurm H, Kostner GM. 1980. Investigation on 2-glycoprotein I in the rat: Isolation from serum and demonstration in lipoprotein density fractions. Int J Biochem 11:265–270.

Roubey RA. 1996. Immunology of the antiphospholipid antibody syndrome. Arthritis Rheum 39:1444–1454.

Shoenfeld Y, Meroni PL. 1992. The beta-2-glycoprotein I and antiphospholipid antibodies. Clin Exp Rheumatol 10:205–209.

Smeenk RJ, Lucassen WA, Swaak TJ. 1987. Is anticardiolipin activity a cross-reaction of anti-DNA or a separate entity? Arthritis Rheum 30:607–617.

Vizi ES, Liang SD, Sperlagh B, Kittel A, Juranyi Z. 1997. Studies on the release and extracellular metabolism of endogenous ATP in rat superior cervical ganglion: Support for neurotransmitter role of ATP. Neuroscience 79:893–903.

Wassef NM, Roerdink F, Swartz Jr, GM, Lyon JA, Berson BJ, Alving CR. 1984. Phosphate-binding specificities of monoclonal antibodies against phosphoinositides in liposomes. Mol Immunol 21:863–868.

Wassef NM, Swartz GM, Alving BM, Alving CR. 1993. ATP specifically bound as a hapten to a monoclonal anti-phospholipid antibody retains phosphate donor activity. Biochem Biophys Res Commun 190:582–588.

